# PET/CT in Oncology: Current Status and Perspectives

**DOI:** 10.1007/s40134-013-0016-x

**Published:** 2013-05-03

**Authors:** Johannes Czernin, Martin Allen-Auerbach, David Nathanson, Ken Herrmann

**Affiliations:** 1 Ahmanson Translational Imaging Division, Department of Molecular and Medical Pharmacology, David Geffen School of Medicine, University of California, Los Angeles, 10833 Le Conte Avenue, Room AR-23-222 CHS, Los Angeles, CA 90095-1782 USA; 2 Department of Nuclear Medicine, Universitätsklinikum Würzburg, Würzburg, Germany

**Keywords:** PET/CT, Oncology, Initial treatment strategies, Subsequent treatment strategies, Molecular imaging

## Abstract

The discovery of the Warburg effect in the early twentieth century followed by the development of the fluorinated glucose analogue ^18^F-fluorodeoxyglucose (^18^F-FDG) and the invention of positron emission tomographs laid the foundation of clinical PET/CT. This review discusses the challenges and obstacles in clinical adoption of this technique. We then discuss advances in instrumentation, including the critically important introduction of PET/CT and current PET/CT protocols. Moreover, we provide evidence for the clinical utility of PET/CT for patient management and its potential impact on patient outcome, and address its cost and cost-effectiveness. Although this review largely focuses on ^18^F-FDG imaging, we also discuss a variety of additional molecular imaging approaches that can be used for cancer phenotyping with PET. Throughout this review we emphasize the critical contributions of CT to the strength of PET/CT.

## Introduction

**Fig. 1:**
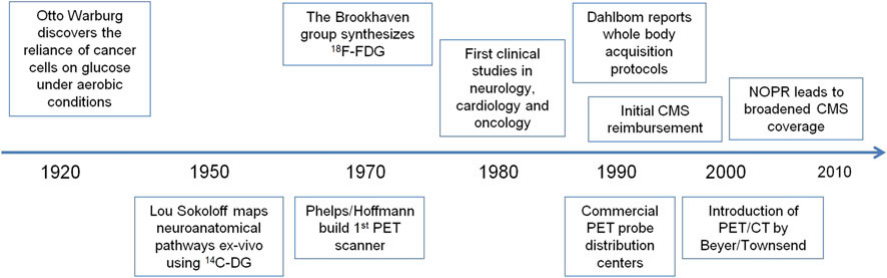
Pivotal events in the history of PET. *CMS* Centers of Medicare and Medicaid Services, ^*14*^ *C-DG* ^14^C-deoxyglucose, ^*18*^ *F-FDG* ^18^F-fluorodeoxyglucose, *NOPR* National Oncologic PET Registry

The foundation of clinical PET/CT was laid by several pivotal events (Fig. 1) that date back as far as the early twentieth century when Otto Warburg [1, 2] discovered that cancer cells switch from oxidative to glucose metabolism even in the presence of oxygen (aerobic glycolysis; Warburg effect). More than three decades later, Luis Sokoloff [3, 4] demonstrated that ^14^C-deoxyglucose autoradiography could be used to map and quantify functional neuroanatomical pathways ex vivo. The translation of this approach became feasible when the fluorinated glucose analogue ^18^F-fluorodeoxyglucose (^18^F-FDG) was developed [5] and Phelps and Hoffmann [6, 7] invented and built the first positron emission tomograph, which made possible the visualization of glycolytic activity in vivo.

Subsequently, ^18^F-FDG and ^13^N-ammonia were used to investigate regional cerebral blood flow and glucose metabolism in patients with epilepsy [8], stroke [9], cancer [10, 11], and dementia [12]. ^18^F-FDG PET for tumor detection was first reported in animal models in 1980 [13], and subsequently in human lung neoplasms in 1987 [14].

PET oncology research accelerated with the development of whole body PET image acquisition protocols [15]. However, clinical adoption was slow, which was explained by (1) the limited number of cyclotrons required for production of PET probes, (2) the practice patterns of oncologists that relied largely on anatomical information for determining initial and subsequent treatment strategies in cancer, and (3) limited or complete lack of reimbursement for clinical PET studies.

The hybrid PET/CT technology introduced by Townsend, Nutt, and Beyer [16] in 1998, the emergence of commercial distribution networks for ^18^F-FDG, together with broadened coverage by the Centers of Medicare and Medicaid Services based on an extensive literature review [17] changed the landscape of cancer imaging. More than 2,500 PET/CT scanners are currently operational in the USA, and the number of clinical PET studies exceeds two million per year.

Given the critically important role of PET/CT in oncology, an appraisal of the current status and perspectives of PET/CT is warranted. Here we discuss advances in PET/CT instrumentation and describe the information that can be derived from the combination of anatomical, functional, and molecular imaging. We then discuss current PET/CT protocols as implemented at our institution and report on initial attempts to standardize image acquisition, reconstruction, and interpretation. We also provide evidence for the clinical utility of PET/CT for patient management and its potential impact on patient outcome and address its cost and cost-effectiveness. Although this review largely focuses on ^18^F-FDG imaging, we will also discuss a variety of additional molecular imaging approaches that can be used for cancer phenotyping with PET. Throughout this review, we emphasize the critical contributions of CT to the strength of PET/CT.

## Advances in Instrumentation

Beyer et al. reported the design of the first PET/CT scanner [16]. Its conceptual advantages included near ideal alignment between PET and CT images and CT-based corrections for photon attenuation [18]. The prototype consisted of a single-detector spiral CT scanner and a half-ring PET scanner. Initial studies conducted at the University of Pittsburgh demonstrated a diagnostic advantage of the hybrid system over PET and CT alone by more accurate lesion localization and improved diagnostic confidence [19].

Since 1998, both the PET component and the CT component of PET/CT systems have improved dramatically. For PET, fast scintillators with high stopping power such as lutetium orthosilicate and gadolinium orthosilicate have become available and have made time-of-flight PET a clinical reality [20, 21]. Routine use of time-of-flight PET led to a significant improvement in lesion detection, especially when images display significant background noise [22]. Smaller detectors resulted in improved spatial resolution. Moreover, high count rate statistics permit image acquisition times as short as 1 min per bed position in some patients [23]. Iterative image reconstruction methods such as ordered subset expectation maximization resulted in further improvements in image quality [24].

At the same time, significant improvements in CT instrumentation occurred. CT devices equipped with 64 detectors are now routinely incorporated in PET/CT. Whole-body anatomical images of high diagnostic quality can thus be acquired in a few seconds and are used for photon attenuation correction while at the same time providing diagnostic information.

## Embedding Tumor Biology in Anatomy

Cancer detection, staging, restaging, and therapy monitoring has traditionally been the domain of anatomical imaging. With deeper insights into tumor biology, the limitations of the anatomical approach have become evident [25••]. For instance, soft tissue masses are composed of viable tumor, necrosis, fibrosis, and inflammation, distinctions that cannot be made on the basis of anatomical assessments. Neither the dignity of the viable tumor component nor its grade or biological behavior can be reliably predicted from the appearance, shape, or size of anatomical masses. Changes in tumor size do not reliably predict tumor responses to therapy. Histologically identical tumors may have very different genotypes and phenotypes [26], an important observation with significant therapeutic implications and consequences.

Yet anatomy provides a highly useful framework within which tumor biology can be studied with PET. Thus, PET and CT contribute equally to the value and strength of PET/CT. For instance, surgical interventions, radiation therapy, or biopsy planning rely on presurgical anatomical imaging studies. However, by more precise cancer staging, PET plays a pivotal role in stratifying patients into those who benefit versus those who do not benefit from surgery [27]. Moreover, target definition and dose painting can be improved by PET prior to radiation therapy [28] and PET can guide interventional radiologists to the most appropriate biopsy site [29].

Assessing tumor responses to therapy in cancer patients is the strength and domain of ^18^F-FDG PET [30]. However, combining metabolic with anatomical information by measuring total lesion glycolysis [31] or total metabolic tumor volume [32, 33] might further improve treatment response predictions.

## Reading Molecular Cancer Signatures with PET

Nearly 100 years ago, Otto Warburg observed that proliferating tumor cells more readily metabolize glucose to lactate despite nonlimiting oxygen conditions. This energy-inefficient process, termed aerobic glycolysis, provides tumors with the ability to rapidly generate the macromolecules required for cell proliferation and growth. The high glucose utilization of cancer cells is enabled through a metabolic rewiring driven by altered signal transduction pathways. For example, mutations in the RAS–MAPK–ERK and PI3K–Akt–mTOR pathways and SRC can induce higher glucose uptake through modulating glucose transporter expression and translocation [34–36]. In addition to its roles in stimulating glucose transport, Akt can also promote hexokinase 1 and phosphofructokinase activity; these are two important enzymes in the production of glycolytic intermediates [37–39]. Furthermore, overexpression of the transcription factors MYC and HIFα, whose expression can be regulated by PI3K–Akt–mTOR, can influence the expression of several genes associated with glycolysis [37]. These include glucose transporters and specific enzymes that promote the aerobic glycolysis phenotype, such as PKM2 and PDK1 [40–42]. Thus, ^18^F-FDG PET images depict the complex interplay among gene expression [43], translation, and various signal transduction pathways (reviewed in [44]). The information extracted from these images can provide insights into tumor proliferative activity, aggressiveness [45, 46], and prognosis [47]. Moreover, ^18^F-FDG PET might be a useful readout of therapeutic interventions targeting one or several of these signal transduction pathways. However, a limitation is the limited specificity of ^18^F-FDG PET owing to physiologic glucose consumption that can occur in benign tissue (e.g., brown fat, colonic and pelvic activity, infection and inflammations, and rebound thymic hyperplasia).

Other metabolic pathways provide nutrients and metabolic building blocks for cancer cells. This is important because alternative metabolic pathways can be exploited for PET of tumors that exhibit low glycolytic activity [48]. For instance, glutamine transport and metabolism, controlled by MYC, is upregulated in many cancers [49]. This may represent an alternative to glucose metabolism, or more likely, a synergistic strategy of cancer cells to generate the energy required for growth and survival.

Increased amino acid transport and metabolism may provide important prognostic information. L-type amino acid transporter 1 (LAT1) expression was correlated with long-term outcome in lung cancer patients [50] and pancreatic cancer patients [51]. Consistently, the degree of ^11^C-methionine uptake was correlated with LAT1 expression in glioblastoma [52], which in turn may provide prognostic information. ^18^F-DOPA has been used to diagnose and grade primary brain tumors [53, 54], and brain tumor recurrence [55] can be readily detected with this PET probe. Increased tumor ^18^F-DOPA concentration in patients with suspected brain tumor recurrence provides important prognostic information [56].

As mentioned above, the PI3K–Akt–mTOR pathway is a key regulator of tumor cell metabolism. In addition to its role in glycolysis, its activation can also promote lipid biosynthesis for cell membrane incorporation [57]. ^11^C-choline and ^11^C-acetate, imaging probes that target choline kinase and fatty acid synthase, respectively, have been used to image increased lipid incorporation into membrane lipid pools in primary and metastatic prostate cancer [58–63] and hepatocellular carcinoma [64]. ^11^C-choline prostate cancer imaging has already been introduced into clinical practice in several centers in Europe [65]. Identifying the exact localization of primary, recurrent, or regionally metastatic prostate cancers and thus making possible targeted interventions requires accurate anatomical assessments, which underscores the importance of hybrid imaging modalities.

Tumor cell proliferation can be imaged with ^18^F-fluorothymidine (^18^F-FLT), a thymidine analogue that enters tumor cells via nucleoside transporters and is phosphorylated by thymidine kinase 1 [66]. It can thus serve as a marker of tumor cell proliferation. Significant correlations between ^18^F-FLT uptake and the expression of the proliferation marker Ki-67 have been demonstrated in lung cancer [67], colorectal cancer [68], hepatocellular carcinoma [69], and other types of cancer (reviewed in [70]). However, changes in tumor ^18^F-FLT uptake in response to treatment were unrelated to histopathological response and Ki-67 expression in soft tissue sarcoma [71, 72]. Thus, various chemotherapies might affect tumor ^18^F-FLT uptake and uncouple it from biological indices of proliferation through a variety of mechanisms [71]. The role of ^18^F-FLT imaging in managing cancer patients thus awaits further clarification.

Links between hormone receptor expression and ^18^F-FDG uptake in breast cancer have been reported. For instance, triple-negative breast cancers that are known to carry a poor prognosis exhibit significantly higher ^18^F-FDG uptake than estrogen and/or progesterone receptor positive tumors [73]. Thus, metabolic phenotyping of cancers with PET might provide important prognostic information.

Estrogen receptor expression can be imaged directly with PET, which permits response predictions to hormonal therapy in breast cancer [74]. Similarly, androgen receptors expressed in primary or metastatic prostate cancers have been imaged with ^18^F-fluorodihydrotestosterone, which binds to their ligand-binding domain [75]. More recently, a fully humanized, radiolabeled antibody against prostate-specific membrane antigen was developed to image intracellular androgen receptor signaling [76, 77].

These imaging probes might be useful for improved noninvasive phenotyping of prostate cancers, which in turn should make possible more individualized therapeutic approaches.

Other receptor-based approaches include the imaging of somatostatin and bombesin receptors in a variety of neuroendocrine tumors and prostate cancer, respectively [78]. Assessing the expression of these receptors in sometimes very small tumor lesions mandates the use of anatomical imaging for exact localization of tracer accumulation.

Highly specific imaging approaches use antibodies, diabodies, or minibodies that target cell surface structures. These molecules can be labeled with diagnostic/therapeutic radioisotope pairs, such as ^64^Cu/^67^Cu, ^86^Y/^90^Y, and ^124^I/^131^I (reviewed in [79]). Thus, theranostic approaches have become feasible and organ tracer distribution in well-defined anatomical volumes (by CT) can provide critically important and accurate dosimetry data for radioimmunotherapy or radiopeptide therapy. The anatomical framework provided by CT is also of pivotal importance for cell trafficking by various PET reporter gene imaging approaches, for instance, when therapeutic cells are dispersed throughout the whole body [80].

In summary, a large and diverse portfolio of molecular PET probes has emerged that can be used for cancer phenotyping by addressing most of the hallmarks of cancer [81••].

## Structural and Functional Information Derived from CT

Tumor size cannot be measured accurately with PET. Such measurements derived from CT (albeit with limitations) are important for T staging and for determining cancer invasion into adjacent tissues [82]. Despite considerable limitations, changes in tumor size are still most frequently used to determine tumor responses to therapy [83], and anatomical information is indispensable for the planning of biopsy, surgery, and radiation therapy.

Yet, CT images might also identify specific tumor phenotypes. For instance, tumor perfusion can be estimated by employing dynamic CT at high temporal resolution. Such measurements require the intravenous administration of contrast agents, the measurement of tissue density before, during, and after contrast agent administration, and the definition of regions of interest, from which the arterial density input function can be derived. With use of kinetic models, the density–time data of the arterial input function and those of the tumor can then be used to estimate tumor perfusion [84]. Such measurements have revealed perfusion differences between normal tissues and cancers, and tumor perfusion rates were significantly correlated with microvascular density and vascular endothelial growth factor expression in lung cancer [85, 86] and pancreatic cancer [87] (reviewed in [84]). These measurements expose patients to considerable levels of ionizing radiation, ranging from 12.3 to 36.7 mSv [88]. However, such measurements are relevant because they (1) may inform about tumor vascularity and angiogenesis and thus provide important prognostic information, (2) provide predictive information about responses to antiangiogenic drugs, and (3) may allow predictions about efficient drug delivery to tumors.

Measurements of tumor heterogeneity or tumor texture may also enhance the information that can be derived from CT images. Differences in texture might reflect variable tissue vascularization. Tumor texture was correlated with the degree of tumor hypoxia and angiogenesis [89]. Methods of texture analysis are also under development for ^18^F-FDG PET [90].

In summary, PET/CT is by far the most mature and comprehensive technology for structural, functional, and molecular phenotyping of cancer at the whole-body level. Its applications are near limitless in oncology and include diagnosing, staging, therapy monitoring, and treatment stratification.

## PET/CT Protocols

**Fig. 2:**
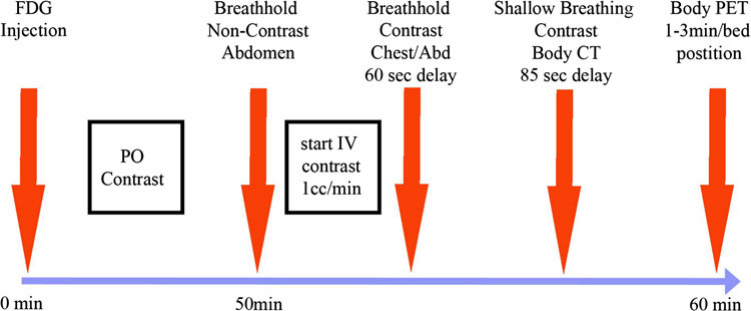
University of California, Los Angeles PET/CT protocol providing a multiphase abdominal CT scan, a breath-hold chest CT scan, and a whole-body contrast CT scan performed during shallow breathing for fusion with the PET images. *IV* intravenous, *PO* per os

PET/CT protocols differ among institutions and countries [91, 92]. PET/CT studies can be performed with or without single-phase or multiphase intravenous and/or oral administration of contrast agent so that fully diagnostic anatomical and molecular whole-body surveys can be obtained. Contrast CT studies for PET/CT appear to result in incremental improvements in diagnostic accuracy in some cancers. At the University of California, Los Angeles, we inject 7.4 mBq of ^18^F-FDG per kilogram intravenously after a 4–6 h fast (Fig. 2). Contrast agent is given orally at the time of the tracer injection. Following a 1-h uptake period, patients are positioned on the scanner table. The scan commences with a breath-hold chest CT scan to identify small lung nodules that may be missed during shallow breathing [93].

Following the breath-hold chest CT scan, we intravenously administer contrast agent using protocols that best address the clinical problem. The feasibility of multiphase contrast protocols for PET/CT has recently been reported [94, 95].

The whole-body contrast CT and PET images are acquired during shallow breathing, which results in acceptable alignment between the PET and CT images [96]. The whole-body contrast CT image is used for diagnostic purposes and for attenuation correction [18]. We use oral and intravenous administration of contrast agents in all patients in whom a stand-alone CT study would employ such protocols.

We use weight-based protocols for PET studies with shorter acquisition times in light patients and longer acquisition times in heavy patients [23]. The total scan times for whole-body PET/CT protocols with intravenous administration of contrast agent average less than 30 min per patient and can be as short as 15 min. Thus, a true “one stop shop” diagnostic imaging approach has become feasible [97].

## Clinical Utility of PET/CT

In 2007 we reported that ^18^F-FDG PET/CT was superior to conventional imaging and PET or CT alone for staging and restaging of most cancers [98]. Subsequent studies confirmed a high staging accuracy of ^18^F-FDG PET/CT in non-small-cell lung cancer [99], breast cancer [100–103], esophageal cancer [104, 105], colorectal cancer [106], lymphoma [107], melanoma [108], cervical cancer [109], head and neck cancers [110], bone and soft tissue sarcomas [111, 112], and myeloma [113].

A recent meta-analysis determined the accuracy of ^18^F-FDG PET/CT for detecting distant metastases or synchronous second cancers in more than 4,300 patients. On the basis of prospectively defined criteria, 41 published studies including patients with primary (*n* = 21 studies) or recurrent (*n* = 14 studies) cancers and patients with primary and recurrent cancers (*n* = 6) were included [114]. In addition, the diagnostic performance of PET/CT was compared with that of conventional imaging in more than 800 patients. Histopathology served as the gold standard in all patients. On a per patient basis, the sensitivity and specificity of PET/CT averaged 93 and 96 %, respectively, which compared favorably with the sensitivity of conventional imaging (52 %). Numerous studies have demonstrated the ability of ^18^F-FDG PET and PET/CT to assess tumor responses to treatment performed as early as after a single cycle of chemotherapy [115], at the middle of chemotherapy, or at the end of chemotherapy [116••, 117, 118].

^18^F-FDG PET/CT has also been investigated as a prognostic marker for outcome predictions. For instance, in 260 patients with Hodgkin’s lymphoma, positive PET findings after two cycles of chemotherapy were associated with a 2-year progression-free survival of 13 %, whereas 95 % of patients with negative PET findings were progression free after 2 years [119]. Other studies confirmed these reports [120]. PET-based risk-adapted therapies have also been shown to be feasible in Hodgkin’s lymphoma [121].

Similar results were reported for non-Hodgkin’s lymphoma [122] and in a variety of solid tumors [123], including cervical cancer [124], soft tissue sarcoma [115, 125] non-small-cell lung cancer [126–128], esophageal cancer [129], breast cancer [130], gastric cancer [131], and other types of cancer (reviewed in [116••, 117]).

Moreover, predictive biomarkers, i.e., those that determine whether therapeutic targets are present, have been developed. These include, among others, androgen receptor imaging [75, 132] and estrogen receptor ligands [74].

In general, tumor responses to neoadjuvant chemo(radiation) therapy correlated with the fraction of necrotic tissue in excised tumors. As a limitation, microscopic residual disease cannot be identified with PET [133].

These results have several implications. First, neoadjuvant therapy follows a predefined treatment protocol and response or nonresponse only becomes evident after surgical removal of the tumors, when changes in therapy can no longer be implemented. Thus, interim ^18^F-FDG PET could determine treatment efficacy early after the start of chemotherapy. Second, unnecessary toxicity of ineffective chemotherapies can be avoided. Third, postsurgical chemotherapies could be tailored on the basis of presurgical treatment responses as determined by PET.

**Fig. 3:**
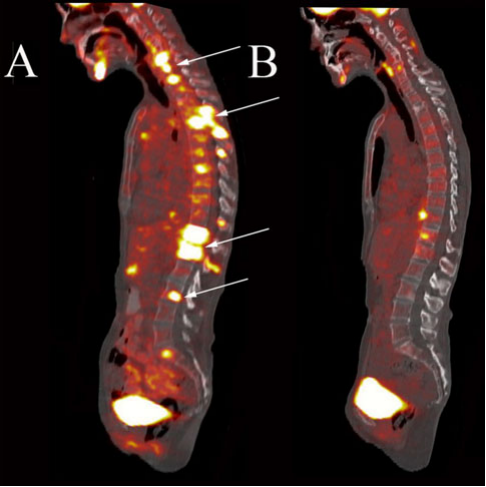
A 54-year-old female patient with non-small-cell lung cancer and extensive metastatic disease to the bones as seen on fused sagittal images (**a**, *arrows*). Two weeks after treatment with the endothelial growth factor receptor inhibitor erlotinib, the bone metastases demonstrate near complete resolution of ^18^F-FDG uptake (**b**)

**Fig. 4:**
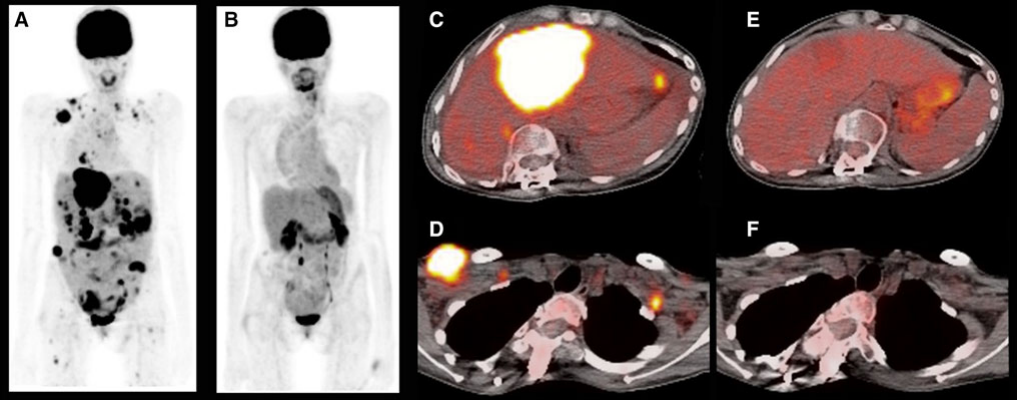
A 26-year-old female patient with metastatic melanoma with PET body maximum intensity projection images at the baseline (**a**) and after 8 weeks of treatment with a B-Raf inhibitor (**b**), demonstrating complete resolution of abnormal ^18^F-FDG uptake. Selected fused axial slices of a pretreatment and posttreatment hepatic lesion (**c**, **e**) and right chest wall lesion (**d**, **f**)

^18^F-FDG PET has also been used successfully to assess tumor responses to targeted, predominantly cytostatic therapies, including imatinib [134, 135], gefitinib [136], erlotinib [137] (Fig. 3), and the B-Raf inhibitor PLX4032 [138] (Fig. 4). The prompt reduction of ^18^F-FDG tumor uptake in response to imatinib and gefitinib appears to be explained by a translocation of membrane-bound glucose transporters into the cytoplasm and thus their inactivation [139]. Moreover, erlotinib increases tumor tissue oxygenation and reduces the tumor uptake of PET hypoxia probes, which may also account for reductions in tumor ^18^F-FDG uptake in response to treatment [140].

Combining anatomical and functional tumor response assessments may further improve treatment response assessments. Standardized uptake values (SUV) or metabolic rates in micromoles per gram per minute describe metabolic activity per gram of tissue but not metabolic rates within the entire tumor volume [141]. This limitation can be overcome with PET/CT by deriving the total lesion glycolysis (i.e., SUV × volume) [31]. These or similar approaches improved tumor response assessments in breast cancer [142] and non-small-cell lung cancer [143] but not in soft tissue sarcoma patients undergoing neoadjuvant therapy [144]. A recent review emphasizes the need for prospective studies to define the value of this integrated approach for tumor treatment response assessments [145].

## Impact of PET on Patient Management

Diagnostic accuracy and the ability to assess tumor responses to treatment are only two possible end points of clinical imaging studies. Other end points include impact on patient management and outcome. The National Oncologic PET Registry, which that now includes more than 300,000 patients [146, 147], provided evidence for a highly significant impact of ^18^F-FDG PET on patient management across a wide variety of cancers. Patient management was affected in more than 30 % of all patients regardless of the study indication. However, the National Oncologic PET Registry did not address the impact of management changes on patient outcome. The challenges to establish such evidence have been summarized recently [148–150].

## Standardization

Any imaging approach must be accurate and reproducible, should provide clinically meaningful diagnostic and prognostic information, and should improve patient management and outcome. As mentioned earlier, a high accuracy of PET/CT for diagnosing, staging, and therapy monitoring has been established. A recent meta-analysis confirmed the good reproducibility of ^18^F-FDG tumor uptake measurements [151].

However, PET/CT protocols are not well standardized, which is a precondition to achieve widespread adoption and acceptance of any diagnostic modality. CT-based RECIST is based on tumor size measurements whereby changes in tumor size are used to define complete response, partial response, and stable or progressive disease in response to therapy [83, 152]. Limitations of the approach have been summarized by Weber [30] and include (1) considerable interobserver variability in size measurements, (2) inaccurate differentiation between viable and nonviable tumor resulting in underestimations of responses; (3) overestimation of responses if tumor regrowth occurs rapidly, (4) inability to differentiate stable disease from beneficial response to (predominantly cytostatic) therapy, and (5) apparent stable disease that denotes slowly growing tumors rather than a beneficial response to treatment. However, although inherently inaccurate [30], RECIST provides simple guidelines for defining response or nonresponse to therapy. It is this simplicity that has led to the widespread adoption of RECIST in clinical practice.

Matters are more complicated for PET. Recent surveys highlight a substantial variability in image acquisition, reconstruction, and analysis, emphasizing the need for standardization in community-based [91] and academic [92] imaging centers.

Boellaard [153] has provided highly useful suggestions for image acquisition, reconstruction, and analysis methods. Young et al. [154] and Wahl et al. [118] have suggested PET-based treatment response criteria. Response criteria in lymphoma therapy have been adopted by many centers [155, 156]. However, no international consensus has been reached, a critically important shortcoming that needs to be addressed urgently.

Demonstrating a beneficial impact of ^18^F-FDG on patient outcome requires large trials with well-defined clinical end points. Over the last few years several such studies have been published. The PETAL trial was performed in lymphoma patients who had positive findings on pretreatment ^18^F-FDG scans [157]. Patients were initially treated with two cycles of CHOP followed by an interim PET scan. Treatment was continued in patients with negative PET findings, whereas those with positive PET findings were randomized to receive six cycles of R-CHOP versus an alternative therapy. Preliminary outcome data after 6 months revealed that relapses occurred almost six times more frequently in patients with positive findings on interim PET scans than in those with negative findings on interim PET scans (17 vs 3 %; *p* = 0.036).

The Municon trial was a nonrandomized study in esophageal cancer patients that used ^18^F-FDG PET findings 2 weeks after the start of treatment to either proceed with (in metabolic responders) or discontinue (in nonresponders) neoadjuvant therapy [158]. Nonresponding patients underwent surgery. The improved survival of metabolic responders underscored the feasibility and value of PET-guided treatment decisions. As another example, lung cancer patients who were randomized to presurgical workup with PET/CT had a significantly lower number of futile surgical procedures than those who underwent conventional staging [99]. Finally, colorectal cancer patients were randomized to either conventional follow-up or ^18^F-FDG PET follow-up [159]. Tumor recurrence was detected significantly earlier in the PET group, and these recurrences were more frequently cured by surgery.

Thus, PET-based risk-adapted therapy approaches are feasible and can affect patient outcome beneficially.

## The Cost (Effectiveness) of PET/CT

Diagnostic tests in cancer have to meet high cost-effectiveness standards. The lack of agreement about basic principles for generating such evidence has recently been reviewed critically [150, 160].

High-end PET/CT systems range in price from $2.5 million to $3.0 million and operational costs including service contracts are substantial. In the USA, ^18^F-FDG PET/CT scans are currently reimbursed by Medicare at $1,150 per scan This is comparable to the reimbursement level of whole-body CT scans. Reimbursement for PET has significantly decreased (by more than 40 %) over the last 10 years. Cancer imaging expenditure accounts for approximately 4.6 % of overall Medicare cancer care costs [161]. Approximately one fifth of this, or around 1 % of Medicare expenditure, was incurred from PET/CT [162]. Although they are increasing at a lower rate, much more significant costs arise from inpatient and outpatient care, cancer drugs, physician services, and hospice care [162]. Moreover, individual procedural costs do not reflect the cost-effectiveness of diagnostic tests. Several studies have suggested that PET/CT is cost-effective across a variety of cancers by improving patient management, which in turn reduces downstream costs incurred because of incorrect management decisions [99, 163–166].

## Future Perspectives

Despite the emergence of PET/MRI [167–169] the role of ^18^F-PET/CT in initial and subsequent patient management decisions will expand over the next decade. PET/MRI is likely to find a role in addressing some specific clinical questions. However, its high cost and operational complexity suggests that its routine clinical use will remain limited. Even currently, the use of MRI in cancer is significantly less than that of CT. For instance, in patients diagnosed with breast, colorectal, lung, and prostate cancer and lymphoma in 2006, CT was used 3.5, 12, 5.5, 4, and 6.3 times more frequently than MRI in the first 2 years after diagnosis [161]. These data are informative as they provide a realistic outlook for the potential use of or market for PET/MRI in cancer.

Concerns about radiation exposure through medical imaging have been raised [170]. Fully diagnostic PET/CT studies may expose patients to radiation doses as high as 25 mSv. However, Brenner and Hall [170] have correctly pointed to the greatly reduced relevance of this perceived risk for patients with limited life expectancy [171]. Furthermore, a recent analysis concluded that “risks of medical imaging at effective doses below 50 mSv for single procedures or 100 mSv for multiple procedures over short time periods are too low to be detectable and may be nonexistent” and that “predictions of hypothetical cancer incidence and deaths in patient populations exposed to such low doses are highly speculative and should be discouraged” because they “are harmful … and may cause some patients and parents to refuse medical imaging procedures” [172].

PET/CT applications in oncology will be refined by standardizing image acquisition, reconstruction, and analysis as well by arriving at internationally accepted treatment response criteria. Highly targeted imaging probes to determine whether therapeutic targets are expressed and active will emerge that will permit treatment stratification and thus individualized therapy approaches. Generator-based production of ^68^Ga permits the labeling of peptides for peptide receptor imaging without the requirement of an on-site cyclotron. This has expanded the use of PET to include neuroendocrine tumors (^68^Ga DOTATATE, ^68^Ga DOTATOC) [173, 174] and for depicting neoangiogenesis using labeled RGD peptides [175]. Moreover, the labeling of bombesin receptor agonists and antagonists shows promise for imaging prostate cancer [176].

Other promising PET approaches include probes that target cell surface antigens, intracellular proteins, and hypoxia. PET reporter gene imaging will be used for trafficking of cell-based therapies. Finally, drug development will be facilitated by PET-based pharmacokinetic and pharmacodynamic studies [177]. At the same time, functional parameters such as tumor perfusion and texture will be derived from dynamic CT images that will provide indices of tumor vascularization and angiogenesis.

In summary, CT in PET/CT provides the anatomical framework within which the biology of cancer can be visualized by PET. This powerful combination will be used to further refine diagnostic, prognostic, intermediate end point, and predictive biomarkers in cancer patients. The role of PET/MRI awaits definition. We believe that in addition to the great potential in brain imaging, the advantage of reduced radiation exposure could lead to wider use in the pediatric population. Finally, cancer patients who undergo MRI studies for cancer assessments might very well benefit from the addition of PET in a single session.

## References

[1] Warburg O, Posener K, Negelein E (1924). The metabolism of cancer cells. Biochem Zeitschr.

[2] Warburg O, Wind F, Negelein E (1927). The metabolism of tumors in the body. J Gen Physiol.

[3] Sokoloff L. The history of neuroscience in autobiography. Washington, D.C: Society of Neuroscience; 1996. p. 1–493.

[4] Sokoloff L, Reivich M, Kennedy C, Des Rosiers M, Patlak C, Pettigrew K (1977). The [14C]deoxyglucose method for the measurement of local cerebral glucose utilization: theory, procedure, and normal values in the conscious and anesthetized albino rat. J Neurochem.

[5] Gallagher B, Fowler J, Gutterson N, MacGregor R, Wan C, Wolf A (1978). Metabolic trapping as a principle of oradiopharmaceutical design: some factors responsible for the biodistribution of [18F] 2-deoxy-2-fluoro-d-glucose. J Nucl Med.

[6] Phelps M, Hoffman E, Mullani N, Ter-Pogossian M (1975). Application of annihilation coincidence detection to transaxial reconstruction tomography. J Nucl Med.

[7] Ter-Pogossian M, Phelps M, Hoffman E, Mullani N (1975). A positron-emission transaxial tomograph for nuclear imaging (PETT). Radiology.

[8] Kuhl D, Engel JJ, Phelps M, Selin C (1980). Epileptic patterns of local cerebral metabolism and perfusion in humans determined by emission computed tomography of 18FDG and 13NH3. Ann Neurol.

[9] Kuhl D, Phelps M, Kowell A, Metter E, Selin C, Winter J (1980). Effects of stroke on local cerebral metabolism and perfusion: mapping by emission computed tomography of 18FDG and 13NH3. Ann Neurol.

[10] Phelps ME, Hoffman EJ, Coleman RE, Welch MJ, Raichle ME, Weiss ES (1976). Tomographic images of blood pool and perfusion in brain and heart. J Nucl Med.

[11] Yen C-K, Yano Y, Budinger TF, Friedland RP, Derenzo SE, Huesman RH (1982). Brain tumor evaluation using Rb-82 and positron emission tomography. J Nucl Med.

[12] Benson D, Kuhl D, Phelps M, Cummings J, Tsai S (1981). Positron emission computed tomography in the diagnosis of dementia. Trans Am Neurol Assoc.

[13] Som P, Atkins H, Bandoypadhyay D, Fowler J, MacGregor R, Matsui K (1980). A fluorinated glucose analog, 2-fluoro-2-deoxy-d-glucose (F-18): nontoxic tracer for rapid tumor detection. J Nucl Med.

[14] Nolop K, Rhodes C, Brudin L, Beaney R, Krausz T, Jones T (1987). Glucose utilization in vivo by human pulmonary neoplasms. Cancer.

[15] Dahlbom M, Hoffman EJ, Hoh CK, Schiepers C, Rosenqvist G, Hawkins RA (1992). Whole-body positron emission tomography: part I. Methods and performance characteristics. J Nucl Med.

[16] Beyer T, Townsend D, Brun T, Kinahan P, Charron M, Roddy R (2000). A combined PET/CT scanner for clinical oncology. J Nucl Med.

[17] Gambhir SS, Czernin J, Schwimmer J, Silverman DHS, Coleman RE, Phelps ME (2001). A tabulated summary of the FDG PET literature. J Nucl Med.

[18] Kinahan P, Townsend D, Beyer T, Sashin D (1998). Attenuation correction for a combined 3D PET/CT scanner. Med Phys.

[19] Martinelli M, Townsend D, Meltzer C, Villemagne V. 7. Survey of results of whole body imaging using the PET/CT at the University of Pittsburgh Medical Center PET Facility. Clin Positron Imaging. 2000;3(4):161. doi:10.1016/S1095-0397(00)00073-X. 10.1016/s1095-0397(00)00073-x 11150764

[20] Mullani N, Markham J, Ter-Pogossian M (1980). Feasibility of time-of-flight reconstruction in positron emission tomography. J Nucl Med.

[21] Budinger T (1983). Time-of-flight positron emission tomography: status relative to conventional PET. J Nucl Med.

[22] Kadrmas DJ, Casey ME, Conti M, Jakoby BW, Lois C, Townsend DW (2009). Impact of time-of-flight on PET tumor detection. J Nucl Med.

[23] Halpern B, Dahlbom M, Quon A, Schiepers C, Waldherr C, Silverman D (2004). Impact of patient weight and emission scan duration on PET/CT image quality and lesion detectability. J Nucl Med.

[24] Hudson H, Larkin R (1994). Accelerated image reconstruction using ordered subsets of projection data. IEEE Trans Med Imaging.

[25] •• Sharma MR, Maitland ML, Ratain MJ. RECIST: no longer the sharpest tool in the oncology clinical trials toolbox—point. Cancer Res. 2012;72(20):5145–9. doi:10.1158/0008-5472.can-12-0058. *This article introduces a new paradigm in which therapeutics are assessed on a continuous scale by evidence of efficacy, using a variety of quantitative tools that take advantage of technologic innovations and increasing understanding of cancer biology.* 10.1158/0008-5472.CAN-12-0058 22952219

[26] Ruiz C, Lenkiewicz E, Evers L, Holley T, Robeson A, Kiefer J (2011). Advancing a clinically relevant perspective of the clonal nature of cancer. Proc Natl Acad Sci USA.

[27] van Tinteren H, Hoekstra OS, Smit EF, van den Bergh JHAM, Schreurs AJM, Stallaert RALM et al. Effectiveness of positron emission tomography in the preoperative assessment of patients with suspected non-small-cell lung cancer: the PLUS multicentre randomised trial. Lancet. 2002;359(9315):1388–92. doi:10.1016/S0140-6736(02)08352-6. 10.1016/s0140-6736(02)08352-6 11978336

[28] Wahl RL, Herman JM, Ford E. The promise and pitfalls of positron emission tomography and single-photon emission computed tomography molecular imaging–guided radiation therapy. Semin Radiat Oncol. 2011;21(2):88–100. doi:10.1016/j.semradonc.2010.11.004. 10.1016/j.semradonc.2010.11.004 PMC4337868 21356477

[29] Tatli S, Gerbaudo VH, Feeley CM, Shyn PB, Tuncali K, Silverman SG. PET/CT-guided percutaneous biopsy of abdominal masses: initial experience. J Vasc Interv Radiol. 2011;22(4):507–14. doi:10.1016/j.jvir.2010.12.035. 10.1016/j.jvir.2010.12.035 21367619

[30] Weber W (2009). Assessing tumor response to therapy. J Nucl Med.

[31] Larson SM, Erdi Y, Akhurst T, Mazumdar M, Macapinlac HA, Finn RD et al. Tumor treatment response based on visual and quantitative changes in global tumor glycolysis using PET-FDG imaging: the visual response score and the change in total lesion glycolysis. Clin Positron Imaging. 1999;2(3):159–71. doi:10.1016/S1095-0397(99)00016-3. 10.1016/s1095-0397(99)00016-3 14516540

[32] Francis RJ, Byrne MJ, van der Schaaf AA, Boucek JA, Nowak AK, Phillips M (2007). Early prediction of response to chemotherapy and survival in malignant pleural mesothelioma using a novel semiautomated 3-dimensional volume-based analysis of serial 18F-FDG PET scans. J Nucl Med.

[33] Lee P, Weerasuriya DK, Lavori PW, Quon A, Hara W, Maxim PG et al. Metabolic tumor burden predicts for disease progression and death in lung cancer. Int J Radiat Oncol Biol Phys. 2007;69(2):328–33. doi:10.1016/j.ijrobp.2007.04.036. 10.1016/j.ijrobp.2007.04.036 17869659

[34] Flier JS, Mueckler MM, Usher P, Lodish HF (1987). Elevated levels of glucose transport and transporter messenger RNA are induced by ras or src oncogenes. Science.

[35] Barthel A, Okino ST, Liao J, Nakatani K, Li J, Whitlock JP (1999). Regulation of GLUT1 gene transcription by the serine/threonine kinase Akt1. J Biol Chem.

[36] Kohn AD, Summers SA, Birnbaum MJ, Roth RA (1996). Expression of a constitutively active Akt Ser/Thr kinase in 3T3-L1 adipocytes stimulates glucose uptake and glucose transporter 4 translocation. J Biol Chem.

[37] Cairns RA, Harris IS, Mak TW (2011). Regulation of cancer cell metabolism. Nat Rev Cancer.

[38] Gottlob K, Majewski N, Kennedy S, Kandel E, Robey RB, Hay N (2001). Inhibition of early apoptotic events by Akt/PKB is dependent on the first committed step of glycolysis and mitochondrial hexokinase. Genes Dev.

[39] Deprez J, Vertommen D, Alessi DR, Hue L, Rider MH (1997). Phosphorylation and activation of heart 6-phosphofructo-2-kinase by protein kinase B and other protein kinases of the insulin signaling cascades. J Biol Chem.

[40] David CJ, Chen M, Assanah M, Canoll P, Manley JL (2010). HnRNP proteins controlled by c-Myc deregulate pyruvate kinase mRNA splicing in cancer. Nature.

[41] Luo W, Semenza GL (2011). Pyruvate kinase M2 regulates glucose metabolism by functioning as a coactivator for hypoxia-inducible factor 1 in cancer cells. Oncotarget.

[42] Cantor JR, Sabatini DM (2012). Cancer cell metabolism: one hallmark, many faces. Cancer Discov.

[43] Nair VS, Gevaert O, Davidzon G, Napel S, Graves EE, Hoang CD (2012). Prognostic PET 18F-FDG uptake imaging features are associated with major oncogenomic alterations in patients with resected non–small cell lung cancer. Cancer Res.

[44] Kelloff GJ, Hoffman JM, Johnson B, Scher HI, Siegel BA, Cheng EY (2005). Progress and promise of FDG-PET imaging for cancer patient management and oncologic drug development. Clin Cancer Res.

[45] Yap CS, Czernin J, Fishbein MC, Cameron RB, Schiepers C, Phelps ME (2006). Evaluation of thoracic tumors with 18F-fluorothymidine and 18F-fluorodeoxyglucose-positron emission tomography. Chest J.

[46] Buck A, Schirrmeister H, Kühn T, Shen C, Kalker T, Kotzerke J (2002). FDG uptake in breast cancer: correlation with biological and clinical prognostic parameters. Eur J Nucl Med.

[47] Vansteenkiste JF, Stroobants SG, Dupont PJ, De Leyn PR, Verbeken EK, Deneffe GJ (1999). Prognostic importance of the standardized uptake value on 18F-fluoro-2-deoxy-glucose–positron emission tomography scan in non–small-cell lung cancer: an analysis of 125 cases. J Clin Oncol.

[48] Lieberman BP, Ploessl K, Wang L, Qu W, Zha Z, Wise DR (2011). PET imaging of glutaminolysis in tumors by 18F-(2S,4R)4-fluoroglutamine. J Nucl Med.

[49] Wisea D, DeBerardinis R, Mancusoa A, Sayeda N, Zhang X, Pfeiffer H (2008). Myc regulates a transcriptional program that stimulates mitochondrial glutaminolysis and leads to glutamine addiction. PNAS.

[50] Kaira K, Oriuchi N, Imai H, Shimizu K, Yanagitani N, Sunaga N et al. Prognostic significance of l-type amino acid transporter 1 (LAT1) and 4F2 heavy chain (CD98) expression in stage I pulmonary adenocarcinoma. Lung Cancer. 2009;66(1):120–6. doi:10.1016/j.lungcan.2008.12.015. 10.1016/j.lungcan.2008.12.015 19171406

[51] Yanagisawa N, Ichinoe M, Mikami T, Nakada N, Hana K, Koizumi W (2012). High expression of L-type amino acid transporter 1 (LAT1) predicts poor prognosis in pancreatic ductal adenocarcinomas. J Clin Pathol.

[52] Okubo S, Zhen H-N, Kawai N, Nishiyama Y, Haba R, Tamiya T (2010). Correlation of l-methyl-11C-methionine (MET) uptake with l-type amino acid transporter 1 in human gliomas. J Neurooncol.

[53] Chen W, Silverman DHS, Delaloye S, Czernin J, Kamdar N, Pope W (2006). 18F-FDOPA PET imaging of brain tumors: comparison study with 18F-FDG PET and evaluation of diagnostic accuracy. J Nucl Med.

[54] Fueger BJ, Czernin J, Cloughesy T, Silverman DH, Geist CL, Walter MA (2010). Correlation of 6-18F-fluoro-l-dopa PET uptake with proliferation and tumor grade in newly diagnosed and recurrent gliomas. J Nucl Med.

[55] Ledezma CJ, Chen W, Sai V, Freitas B, Cloughesy T, Czernin J et al. 18F-FDOPA PET/MRI fusion in patients with primary/recurrent gliomas: Initial experience. Eur J Radiol. 2009;71(2):242–8. doi:10.1016/j.ejrad.2008.04.018. 10.1016/j.ejrad.2008.04.018 18511228

[56] Walter F, Cloughesy T, Walter MA, Lai A, Nghiemphu P, Wagle N (2012). Impact of 3,4-dihydroxy-6-18F-fluoro-l-phenylalanine PET/CT on managing patients with brain tumors: the referring physician’s perspective. J Nucl Med.

[57] DeBerardinis RJ, Lum JJ, Hatzivassiliou G, Thompson CB. The biology of cancer: metabolic reprogramming fuels cell growth and proliferation. Cell Metab. 2008;7(1):11–20. doi:10.1016/j.cmet.2007.10.002. 10.1016/j.cmet.2007.10.002 18177721

[58] Seltzer MA, Barbaric Z, Belldegrun A, Naitoh J, Dorey F, Phelps ME et al. Comparison of helical computerized tomography, positron emission tomography and monoclonal antibody scans for evaluation of lymph node metastases in patients with prostate specific antigen relapse after treatment for localized prostate cancer. J Urol. 1999;162(4):1322–8. doi:10.1016/S0022-5347(05)68277-8. 10492189

[59] Schöder H, Larson SM. Positron emission tomography for prostate, bladder, and renal cancer. Semin Nucl Med. 2004;34(4):274–92. doi:10.1053/j.semnuclmed.2004.06.004. 10.1053/j.semnuclmed.2004.06.004 15493005

[60] Hara T, Kosaka N, Kishi H (1998). PET imaging of prostate cancer using carbon-11-choline. J Nucl Med.

[61] de Jong IJ, Pruim J, Elsinga PH, Vaalburg W, Mensink HJA. Visualization of prostate cancer with 11C-choline positron emission tomography. Eur Urol. 2002;42(1):18–23. doi:10.1016/S0302-2838(02)00129-X. 10.1016/s0302-2838(02)00129-x 12121724

[62] Czernin J, Benz M, Allen-Auerbach M (2009). PET imaging of prostate cancer using C-acetate. PET Clin.

[63] Beheshti M, Imamovic L, Broinger G, Vali R, Waldenberger P, Stoiber F (2010). 18F choline PET/CT in the preoperative staging of prostate cancer in patients with intermediate or high risk of extracapsular disease: a prospective study of 130 patients. Radiology.

[64] Ho C-L, Yu SCH, Yeung DWC (2003). 11C-acetate pet imaging in hepatocellular carcinoma and other liver masses. J Nucl Med.

[65] Langsteger W, Heinisch M, Fogelman I. The role of fluorodeoxyglucose, 18F-dihydroxyphenylalanine, 18F-choline, and 18F-fluoride in bone imaging with emphasis on prostate and breast. Semin Nucl Med. 2006;36(1):73–92. doi:10.1053/j.semnuclmed.2005.09.002. 10.1053/j.semnuclmed.2005.09.002 16356797

[66] Shields A, Grierson J, Dohmen B, Machulla H, Stayanoff J, Lawhorn-Crews J (1998). Imaging proliferation in vivo with [F-18]FLT and positron emission tomography. Nat Med.

[67] Vesselle H, Grierson J, Muzi M, Pugsley JM, Schmidt RA, Rabinowitz P (2002). In vivo validation of 3′deoxy-3′-[18F]fluorothymidine ([18F]FLT) as a proliferation imaging tracer in humans: correlation of [18F]FLT uptake by positron emission tomography with Ki-67 immunohistochemistry and flow cytometry in human lung tumors. Clin Cancer Res.

[68] Francis DL, Freeman A, Visvikis D, Costa DC, Luthra SK, Novelli M (2003). In vivo imaging of cellular proliferation in colorectal cancer using positron emission tomography. Gut.

[69] Eckel F, Herrmann K, Schmidt S, Hillerer C, Wieder HA, Krause B-J (2009). Imaging of proliferation in hepatocellular carcinoma with the in vivo marker 18F-fluorothymidine. J Nucl Med.

[70] Chalkidou A, Landau DB, Odell EW, Cornelius VR, O’Doherty MJ, Marsden PK. Correlation between Ki-67 immunohistochemistry and 18F-fluorothymidine uptake in patients with cancer: a systematic review and meta-analysis. Eur J Cancer. 2012;48(18):3499–513. doi:10.1016/j.ejca.2012.05.001. 10.1016/j.ejca.2012.05.001 22658807

[71] Benz MR, Czernin J, Allen-Auerbach MS, Dry SM, Sutthiruangwong P, Spick C (2012). 3′-Deoxy-3′-[18F]fluorothymidine positron emission tomography for response assessment in soft tissue sarcoma. Cancer.

[72] Wieder H, Geinitz H, Rosenberg R, Lordick F, Becker K, Stahl A (2007). PET imaging with [18F]3′-deoxy-3′-fluorothymidine for prediction of response to neoadjuvant treatment in patients with rectal cancer. Eur J Nucl Med.

[73] Koolen BB, Vrancken Peeters MJTFD, Wesseling J, Lips EH, Vogel WV, Aukema TS et al. Association of primary tumour FDG uptake with clinical, histopathological and molecular characteristics in breast cancer patients scheduled for neoadjuvant chemotherapy. Eur J Nucl Med. 2012;39(12):1830–8. doi:10.1007/s00259-012-2211-z. 10.1007/s00259-012-2211-z 22895862

[74] Linden HM, Stekhova SA, Link JM, Gralow JR, Livingston RB, Ellis GK (2006). Quantitative fluoroestradiol positron emission tomography imaging predicts response to endocrine treatment in breast cancer. J Clin Oncol.

[75] Liu A, Dence C, Welch M, Katzenellenbogen J (1992). Fluorine-18-labeled androgens: radiochemical synthesis and tissue distribution studies on six fluorine-substituted androgens, potential imaging agents for prostatic cancer. J Nucl Med.

[76] Evans MJ, Smith-Jones PM, Wongvipat J, Navarro V, Kim S, Bander NH (2011). Noninvasive measurement of androgen receptor signaling with a positron-emitting radiopharmaceutical that targets prostate-specific membrane antigen. Proc Natl Acad Sci USA.

[77] Afshar-Oromieh A, Malcher A, Eder M, Eisenhut M, Linhart HG, Hadaschik BA et al. PET imaging with a [68 Ga]gallium-labelled PSMA ligand for the diagnosis of prostate cancer: biodistribution in humans and first evaluation of tumour lesions. Eur J Nucl Med. 2012:1–10. doi:10.1007/s00259-012-2298-2. 10.1007/s00259-012-2298-2 23179945

[78] Ambrosini V, Fani M, Fanti S, Forrer F, Maecke H (2011). Radiopeptide imaging and therapy in Europe. J Nucl Med.

[79] Wu AM (2009). Antibodies and antimatter: the resurgence of immuno-PET. J Nucl Med.

[80] Yaghoubi S, Campbell D, Radu C, Czernin J (2012). Positron emission tomography reporter genes and reporter probes: gene and cell therapy applications. Theranostics.

[81] •• Hanahan D, Weinberg Robert A. Hallmarks of cancer: the next generation. Cell. 2011;144(5):646–74. doi:10.1016/j.cell.2011.02.013. *This review discusses the conceptual progress of the previously published hallmarks of cancer by adding two emerging hallmarks of potential generality to the existing list: reprogramming of energy metabolism and evading immune destruction.* 10.1016/j.cell.2011.02.013 21376230

[82] Lardinois D, Weder W, Hany TF, Kamel EM, Korom S, Seifert B (2003). Staging of non–small-cell lung cancer with integrated positron-emission tomography and computed tomography. N Engl J Med.

[83] Eisenhauer EA, Therasse P, Bogaerts J, Schwartz LH, Sargent D, Ford R et al. New response evaluation criteria in solid tumours: revised RECIST guideline (version 1.1). Eur J Cancer. 2009;45(2):228–47. doi:10.1016/j.ejca.2008.10.026. 10.1016/j.ejca.2008.10.026 19097774

[84] Petralia G, Bonello L, Viotti S, Preda L, d’Andrea G, Bellomi M (2012). CT perfusion in oncology: how to do it. Cancer Imaging.

[85] Ma S-H, Le H-B, Jia B-H, Wang Z-X, Xiao Z-W, Cheng X-L (2008). Peripheral pulmonary nodules: relationship between multi-slice spiral CT perfusion imaging and tumor angiogenesis and VEGF expression. BMC Cancer.

[86] Li Y, Yang Z-G, Chen T-W, Chen H-J, Sun J-Y, Lu Y-R. Peripheral lung carcinoma: correlation of angiogenesis and first-pass perfusion parameters of 64-detector row CT. Lung Cancer. 2008;61(1):44–53. doi:10.1016/j.lungcan.2007.10.021. 10.1016/j.lungcan.2007.10.021 18055062

[87] d’Assignies G, Couvelard A, Bahrami S, Vullierme M-P, Hammel P, Hentic O (2009). Pancreatic endocrine tumors: tumor blood flow assessed with perfusion CT reflects angiogenesis and correlates with prognostic factors1. Radiology.

[88] Goh V, Dattani M, Farwell J, Shekhdar J, Tam E, Patel S (2011). Radiation dose from volumetric helical perfusion CT of the thorax, abdomen or pelvis. Eur Radiol.

[89] Ganeshan B, Goh V, Mandeville HC, Ng QS, Hoskin PJ, Miles KA (2013). Non–small cell lung cancer: histopathologic correlates for texture parameters at CT. Radiology.

[90] Chicklore S, Goh V, Siddique M, Roy A, Marsden P, Cook GR (2013). Quantifying tumour heterogeneity in 18F-FDG PET/CT imaging by texture analysis. Eur J Nucl Med.

[91] Beyer T, Czernin J, Freudenberg LS (2011). Variations in clinical PET/CT operations: results of an international survey of active PET/CT users. J Nucl Med.

[92] Graham MM, Badawi RD, Wahl RL (2011). Variations in PET/CT methodology for oncologic imaging at U.S. academic medical centers: an imaging response assessment team survey. J Nucl Med.

[93] Allen-Auerbach M, Yeom K, Park J, Phelps M, Czernin J (2006). Standard PET/CT of the chest during shallow breathing is inadequate for comprehensive staging of lung cancer. J Nucl Med.

[94] Aschoff P, Plathow C, Beyer T, Lichy M, Erb G, Öksüz M (2012). Multiphase contrast-enhanced CT with highly concentrated contrast agent can be used for PET attenuation correction in integrated PET/CT imaging. Eur J Nucl Med.

[95] Ippolito D, Capraro C, Guerra L, Ponti E, Messa C, Sironi S (2013). Feasibility of perfusion CT technique integrated into conventional 18FDG/PET-CT studies in lung cancer patients: clinical staging and functional information in a single study. Eur J Nucl Med.

[96] Goerres G, Burger C, Schwitter M, Heidelberg T, Seifert B, von Schulthess G (2003). PET/CT of the abdomen: optimizing the patient breathing pattern. Eur Radiol.

[97] Czernin J, Benz M, Allen-Auerbach M. PET/CT imaging: The incremental value of assessing the glucose metabolic phenotype and the structure of cancers in a single examination. Eur J Radiol. 2010;73(3):470–80. doi:10.1016/j.ejrad.2009.12.023. 10.1016/j.ejrad.2009.12.023 20097498

[98] Czernin J, Allen-Auerbach M, Schelbert HR (2007). Improvements in cancer staging with PET/CT: literature-based evidence as of September 2006. J Nucl Med.

[99] Fischer B, Lassen U, Mortensen J, Larsen S, Loft A, Bertelsen A (2009). Preoperative staging of lung cancer with combined PET–CT. N Engl J Med.

[100] Brennan ME, Houssami N. Evaluation of the evidence on staging imaging for detection of asymptomatic distant metastases in newly diagnosed breast cancer. Breast. 2012;21(2):112–23. doi:10.1016/j.breast.2011.10.005. 10.1016/j.breast.2011.10.005 22094116

[101] Groheux D, Giacchetti S, Delord M, Hindié E, Vercellino L, Cuvier C et al. 18F-FDG PET/CT in staging patients with locally advanced or inflammatory breast cancer: comparison to conventional staging. J Nucl Med. 2013;54(1):5–11. doi:10.2967/jnumed.112.106864. 10.2967/jnumed.112.106864 23213197

[102] Koolen B, Vrancken Peeters M-JFD, Aukema T, Vogel W, Oldenburg HA, Hage J (2012). 18F-FDG PET/CT as a staging procedure in primary stage II and III breast cancer: comparison with conventional imaging techniques. Breast Cancer Res Treat.

[103] Riegger C, Herrmann J, Nagarajah J, Hecktor J, Kuemmel S, Otterbach F (2012). Whole-body FDG PET/CT is more accurate than conventional imaging for staging primary breast cancer patients. Eur J Nucl Med.

[104] Barber TW, Duong CP, Leong T, Bressel M, Drummond EG, Hicks RJ (2012). 18F-FDG PET/CT has a high impact on patient management and provides powerful prognostic stratification in the primary staging of esophageal cancer: a prospective study with mature survival data. J Nucl Med.

[105] Schreurs LMA, Janssens ACJW, Groen H, Fockens P, Dullemen HM, Berge Henegouwen MI et al. Value of EUS in determining curative resectability in reference to CT and FDG-PET: the optimal sequence in preoperative staging of esophageal cancer? Ann Surg Oncol. 2011;1–8. doi:10.1245/s10434-011-1738-8. 10.1245/s10434-011-1738-8 PMC5149559 21547703

[106] Soyka JD, Veit-Haibach P, Strobel K, Breitenstein S, Tschopp A, Mende KA (2008). Staging pathways in recurrent colorectal carcinoma: is contrast-enhanced 18F-FDG PET/CT the diagnostic tool of choice?. J Nucl Med.

[107] El-Galaly TC, d’Amore F, Mylam KJ, de Nully Brown P, Bøgsted M, Bukh A (2012). Routine bone marrow biopsy has little or no therapeutic consequence for positron emission tomography/computed tomography–staged treatment-naive patients with Hodgkin lymphoma. J Clin Oncol.

[108] Reinhardt MJ, Joe AY, Jaeger U, Huber A, Matthies A, Bucerius J (2006). Diagnostic performance of whole body dual modality 18F-FDG PET/CT imaging for N- and M-staging of malignant melanoma: experience with 250 consecutive patients. J Clin Oncol.

[109] Choi HJ, Roh JW, Seo S-S, Lee S, Kim J-Y, Kim S-K (2006). Comparison of the accuracy of magnetic resonance imaging and positron emission tomography/computed tomography in the presurgical detection of lymph node metastases in patients with uterine cervical carcinoma. Cancer.

[110] Mak D, Corry J, Lau E, Rischin D, Hicks RJ (2011). Role of FDG-PET/CT in staging and follow-up of head and neck squamous cell carcinoma. Q J Nucl Med Mol Imaging.

[111] Benz M, Tchekmedyian N, Eilber F, Federman N, Czernin J, Tap W (2009). Utilization of positron emission tomography in the management of patients with sarcoma. Curr Opin Oncol.

[112] Benz MR, Dry SM, Eilber FC, Allen-Auerbach MS, Tap WD, Elashoff D (2010). Correlation between glycolytic phenotype and tumor grade in soft-tissue sarcomas by 18F-FDG PET. J Nucl Med.

[113] van Lammeren-Venema D, Regelink J, Riphagen II, Zweegman S, Hoekstra O, Zijlstra J (2012). 18F-fluoro-deoxyglucose positron emission tomography in assessment of myeloma-related bone disease: a systematic review. Cancer.

[114] Xu G, Zhao L, He Z (2012). Performance of whole-body PET/CT for the detection of distant malignancies in various cancers: a systematic review and meta-analysis. J Nucl Med.

[115] Benz MR, Czernin J, Allen-Auerbach MS, Tap WD, Dry SM, Elashoff D (2009). FDG-PET/CT imaging predicts histopathologic treatment responses after the initial cycle of neoadjuvant chemotherapy in high-grade soft-tissue sarcomas. Clin Cancer Res.

[116] •• Herrmann K, Benz M, Krause B, Pomykala K, Buck A, Czernin J. (18)F-FDG-PET/CT in evaluating response to therapy in solid tumors: where we are and where we can go. Q J Nucl Med Mol Imaging. 2011;55:620–32. *This article provides an overview of the utility of *^*18*^*F-FDG PET/CT for early monitoring of cancer therapy and addresses current and future challenges for its more widespread adoption.* 22231582

[117] Plathow C, Weber WA (2008). Tumor cell metabolism imaging. J Nucl Med.

[118] Wahl RL, Jacene H, Kasamon Y, Lodge MA (2009). From RECIST to PERCIST: evolving considerations for PET response criteria in solid tumors. J Nucl Med.

[119] Gallamini A, Hutchings M, Rigacci L, Specht L, Merli F, Hansen M (2007). Early interim 2-[18F]fluoro-2-deoxy-d-glucose positron emission tomography is prognostically superior to international prognostic score in advanced-stage Hodgkin’s Lymphoma: a report from a joint Italian-Danish study. J Clin Oncol.

[120] Kirby A, Mikhaeel N (2007). The role of FDG PET in the management of lymphoma: what is the evidence base?. Nucl Med Commun.

[121] Dann E (2012). PET/CT adapted therapy in Hodgkin disease: current state of the art and future directions. Curr Oncol Rep.

[122] Casasnovas R-O, Meignan M, Berriolo-Riedinger A, Itti E, Huglo D, Haioun C (2012). Early interim PET scans in diffuse large B-cell lymphoma: can there be consensus about standardized reporting, and can PET scans guide therapy choices?. Curr Hematol Malig Rep.

[123] Weber WA (2006). Positron emission tomography as an imaging biomarker. J Clin Oncol.

[124] Schwarz J, Siegel B, Dehdashti F, Grigsby P (2007). Association of posttherapy positron emission tomography with tumor response and survival in cervical carcinoma. J Am Med Assoc.

[125] Evilevitch V, Weber WA, Tap WD, Allen-Auerbach M, Chow K, Nelson SD (2008). Reduction of glucose metabolic activity is more accurate than change in size at predicting histopathologic response to neoadjuvant therapy in high-grade soft-tissue sarcomas. Clin Cancer Res.

[126] Weber WA, Petersen V, Schmidt B, Tyndale-Hines L, Link T, Peschel C (2003). Positron emission tomography in non-small-cell lung cancer: prediction of response to chemotherapy by quantitative assessment of glucose use. J Clin Oncol.

[127] Hoekstra CJ, Stroobants SG, Smit EF, Vansteenkiste J, van Tinteren H, Postmus PE (2005). Prognostic relevance of response evaluation using [18F]-2-fluoro-2-deoxy-d-glucose positron emission tomography in patients with locally advanced non-small-cell lung cancer. J Clin Oncol.

[128] de Geus-Oei L-F, van der Heijden HFM, Visser EP, Hermsen R, van Hoorn BA, Timmer-Bonte JNH (2007). Chemotherapy response evaluation with 18F-FDG PET in patients with non-small cell lung cancer. J Nucl Med.

[129] Ott K, Weber WA, Lordick F, Becker K, Busch R, Herrmann K (2006). Metabolic imaging predicts response, survival, and recurrence in adenocarcinomas of the esophagogastric junction. J Clin Oncol.

[130] Rousseau C, Devillers A, Sagan C, Ferrer L, Bridji B, Campion L (2006). Monitoring of early response to neoadjuvant chemotherapy in stage II and III breast cancer by [18F]fluorodeoxyglucose positron emission tomography. J Clin Oncol.

[131] Ott K, Herrmann K, Lordick F, Wieder H, Weber WA, Becker K (2008). Early metabolic response evaluation by fluorine-18 fluorodeoxyglucose positron emission tomography allows in vivo testing of chemosensitivity in gastric cancer: long-term results of a prospective study. Clin Cancer Res.

[132] Beattie BJ, Smith-Jones PM, Jhanwar YS, Schoder H, Schmidtlein CR, Morris MJ (2010). Pharmacokinetic assessment of the uptake of 16beta-18F-fluoro-5alpha-dihydrotestosterone (FDHT) in prostate tumors as measured by PET. J Nucl Med.

[133] Tan MB, Linehan D, Hawkins W, Siegel B, Strasberg S (2007). Chemotherapy-induced normalization of FDG uptake by colorectal liver metastases does not usually indicate complete pathologic response. J Gastrointest Surg..

[134] Van den Abbeele A, Badawi R. Use of positron emission tomography in oncology and its potential role to assess response to imatinib mesylate therapy in gastrointestinal stromal tumors (GISTs). Eur J Cancer. 2002;38 Suppl 5:S60–5. 10.1016/s0959-8049(02)80604-9 12528774

[135] Stroobants S, Goeminne J, Seegers M, Dimitrijevic S, Dupont P, Nuyts J et al. 18FDG-positron emission tomography for the early prediction of response in advanced soft tissue sarcoma treated with imatinib mesylate (Glivec^®^). Eur J Cancer. 2003;39(14):2012–20. doi:10.1016/S0959-8049(03)00073-X. 10.1016/s0959-8049(03)00073-x 12957455

[136] Sunaga N, Oriuchi N, Kaira K, Yanagitani N, Tomizawa Y, Hisada T et al. Usefulness of FDG-PET for early prediction of the response to gefitinib in non-small cell lung cancer. Lung Cancer. 2008;59(2):203–10. doi:10.1016/j.lungcan.2007.08.012. 10.1016/j.lungcan.2007.08.012 17913282

[137] Benz MR, Herrmann K, Walter F, Garon EB, Reckamp KL, Figlin R (2011). 18F-FDG PET/CT for monitoring treatment responses to the epidermal growth factor receptor inhibitor erlotinib. J Nucl Med.

[138] Flaherty KT, Puzanov I, Kim KB, Ribas A, McArthur GA, Sosman JA (2010). Inhibition of mutated, activated BRAF in metastatic melanoma. N Engl J Med.

[139] Su H, Bodenstein C, Dumont RA, Seimbille Y, Dubinett S, Phelps ME (2006). Monitoring tumor glucose utilization by positron emission tomography for the prediction of treatment response to epidermal growth factor receptor kinase inhibitors. Clin Cancer Res.

[140] Solomon B, Binns D, Roselt P, Weibe LI, McArthur GA, Cullinane C (2005). Modulation of intratumoral hypoxia by the epidermal growth factor receptor inhibitor gefitinib detected using small animal PET imaging. Mol Cancer Ther.

[141] Weber WA, Figlin R (2007). Monitoring cancer treatment with PET/CT: does it make a difference?. J Nucl Med.

[142] Hatt M, Groheux D, Martineau A, Espié M, Hindié E, Giacchetti S et al. Comparison between 18F-FDG PET image–derived indices for early prediction of response to neoadjuvant chemotherapy in breast cancer. J Nucl Med. 2013;54(3):341–9. doi:10.2967/jnumed.112.108837. 10.2967/jnumed.112.108837 23327900

[143] Soussan M, Chouahnia K, Maisonobe J-A, Boubaya M, Eder V, Morère J-F et al. Prognostic implications of volume-based measurements on FDG PET/CT in stage III non-small-cell lung cancer after induction chemotherapy. Eur J Nucl Med. 2013;1–9. doi:10.1007/s00259-012-2321-7. 10.1007/s00259-012-2321-7 23306807

[144] Benz MR, Allen-Auerbach MS, Eilber FC, Chen HJJ, Dry S, Phelps ME (2008). Combined assessment of metabolic and volumetric changes for assessment of tumor response in patients with soft-tissue sarcomas. J Nucl Med.

[145] Wiele C, Kruse V, Smeets P, Sathekge M, Maes A (2013). Predictive and prognostic value of metabolic tumour volume and total lesion glycolysis in solid tumours. Eur J Nucl Med.

[146] Hillner BE, Siegel BA, Liu D, Shields AF, Gareen IF, Hanna L (2008). Impact of positron emission tomography/computed tomography and positron emission tomography (PET) alone on expected management of patients with cancer: initial results from the national oncologic PET registry. J Clin Oncol.

[147] Hillner BE, Siegel BA, Shields AF, Liu D, Gareen IF, Hunt E (2008). Relationship between cancer type and impact of PET and PET/CT on intended management: findings of the national oncologic PET registry. J Nucl Med.

[148] Weber WA (2011). Is there evidence for evidence-based medical imaging?. J Nucl Med.

[149] Ware RE, Hicks RJ (2011). Doing more harm than good? Do systematic reviews of PET by health technology assessment agencies provide an appraisal of the evidence that is closer to the truth than the primary data supporting its use?. J Nucl Med.

[150] Vach W, Carlsen H, Flemming P, Gerke O, Weber W (2011). Generating evidence for clinical benefit of PET/CT in diagnosing cancer patients. J Nucl Med.

[151] de Langen AJ, Vincent A, Velasquez LM, van Tinteren H, Boellaard R, Shankar LK (2012). Repeatability of 18F-FDG uptake measurements in tumors: a metaanalysis. J Nucl Med.

[152] Costello C, Chuang H, Madewell J (2010). Cancer response criteria and bone metastases: rECIST 1.1, MDA and PERCIST J. Cancer.

[153] Boellaard R (2011). Need for standardization of 18F-FDG PET/CT for treatment response assessments. J Nucl Med.

[154] Young H, Baum R, Cremerius U, Herholz K, Hoekstra O, Lammertsma A (1999). Measurement of clinical and subclinical tumour response using [18F]-fluorodeoxyglucose and positron emission tomography: review and 1999 EORTC recommendations. Eur J Cancer.

[155] Juweid ME, Stroobants S, Hoekstra OS, Mottaghy FM, Dietlein M, Guermazi A (2007). Use of positron emission tomography for response assessment of lymphoma: consensus of the imaging subcommittee of international harmonization project in lymphoma. J Clin Oncol.

[156] Cheson BD, Pfistner B, Juweid ME, Gascoyne RD, Specht L, Horning SJ (2007). Revised response criteria for malignant lymphoma. J Clin Oncol.

[157] Dührsen U, Hüttmann A, Jöckel K-H, Müller S (2009). Positron emission tomography guided therapy of aggressive non-Hodgkin lymphomas—the PETAL trial. Leuk Lymphoma.

[158] Lordick F, Ott K, Krause B-J, Weber WA, Becker K, Stein HJ et al. PET to assess early metabolic response and to guide treatment of adenocarcinoma of the oesophagogastric junction: the MUNICON phase II trial. Lancet Oncol. 2007;8(9):797–805. doi:10.1016/S1470-2045(07)70244-9. 10.1016/S1470-2045(07)70244-9 17693134

[159] Sobhani I, Tiret E, Lebtahi R, Aparicio T, Itti E, Montravers F (2008). Early detection of recurrence by 18FDG-PET in the follow-up of patients with colorectal cancer. Br J Cancer.

[160] Tinteren H, Hoekstra O, Boers M (2004). Do we need randomised trials to evaluate diagnostic procedures?. Eur J Nucl Med.

[161] Dinan M, Curtis L, Hammill B, Patz EJ, Abernethy A, Shea A (2010). Changes in the use and costs of diagnostic imaging among medicare beneficiaries with cancer, 1999–2006. J Am Med Assoc.

[162] Yang Y, Czernin J (2011). Contribution of imaging to cancer care costs. J Nucl Med.

[163] Buck AK, Herrmann K, Stargardt T, Dechow T, Krause BJ, Schreyögg J (2010). Economic evaluation of PET and PET/CT in oncology: evidence and methodologic approaches. J Nucl Med.

[164] Park K, Schwimmer J, Shepherd J, Phelps M, Czernin J, Schiepers C (2001). Decision analysis for the cost-effective management of recurrent colorectal cancer. Ann Surg.

[165] Søgaard R, Fischer B, Mortensen J, Højgaard L, Lassen U (2011). Preoperative staging of lung cancer with PET/CT: cost-effectiveness evaluation alongside a randomized controlled trial. Eur J Nucl Med.

[166] Verboom P, Tinteren H, Hoekstra O, Smit E, Bergh J, Schreurs A (2003). Cost-effectiveness of FDG-PET in staging non-small cell lung cancer: the PLUS study. Eur J Nucl Med.

[167] Drzezga A, Souvatzoglou M, Eiber M, Beer AJ, Fürst S, Martinez-Möller A (2012). First clinical experience with integrated whole-body PET/MR: comparison to PET/CT in patients with oncologic diagnoses. J Nucl Med.

[168] Judenhofer MS, Wehrl HF, Newport DF, Catana C, Siegel SB, Becker M et al. Simultaneous PET-MRI: a new approach for functional and morphological imaging. Nat Med. 2008;14(4):459–65. doi:10.1038/nm1700. 10.1038/nm1700 18376410

[169] Shao Y, Cherry S, Farahani K, Slates R, Silverman R, Meadors K (1997). Development of a PET detector system compatible with MRI/NMR systems. IEEE Trans Nucl Sci.

[170] Brenner D, Hall E (2007). Computed tomography—an increasing source of radiation exposure. N Engl J Med.

[171] Brenner DJ, Shuryak I, Einstein AJ (2011). Impact of reduced patient life expectancy on potential cancer risks from radiologic imaging. Radiology.

[172] Shah DJ, Sachs RK, Wilson DJ (2012). Radiation-induced cancer: a modern view. Br J Radiol.

[173] Maecke HR, Reubi JC (2011). Somatostatin receptors as targets for nuclear medicine imaging and radionuclide treatment. J Nucl Med.

[174] Reubi JC, Maecke HR (2008). Peptide-based probes for cancer imaging. J Nucl Med.

[175] Gaertner FC, Kessler H, Wester HJ, Schwaiger M, Beer AJ (2012). Radiolabelled RGD peptides for imaging and therapy. Eur J Nucl Med Mol Imaging.

[176] Honer M, Mu L, Stellfeld T, Graham K, Martic M, Fischer CR (2011). 18F-labeled bombesin analog for specific and effective targeting of prostate tumors expressing gastrin-releasing peptide receptors. J Nucl Med.

[177] Jones T, Price P (2012). Development and experimental medicine applications of PET in oncology: a historical perspective. Lancet Oncol.

